# Low Cycle Fatigue Life Assessment Based on the Accumulated Plastic Strain Energy Density

**DOI:** 10.3390/ma14092372

**Published:** 2021-05-02

**Authors:** Yifeng Hu, Junping Shi, Xiaoshan Cao, Jinju Zhi

**Affiliations:** Department of Engineering Mechanics, Xi’an University of Technology, Xi’an 710048, China; shjp@xaut.edu.cn (J.S.); caoxsh@xaut.edu.cn (X.C.); zhijinju@126.com (J.Z.)

**Keywords:** accumulated plastic strain energy density, low-cycle fatigue, modified Ramberg-Osgood model, fatigue life assessment

## Abstract

The accumulated plastic strain energy density at a dangerous point is studied to estimate the low cycle fatigue life that is composed of fatigue initiation life and fatigue crack propagation life. The modified Ramberg–Osgood constitutive relation is applied to characterize the stress–strain relationship of the strain-hardening material. The plastic strain energy density under uni-axial tension and cyclic load are derived, which are used as threshold and reference values, respectively. Then, a framework to assess the lives of fatigue initiation and fatigue crack propagation by accumulated plastic strain energy density is proposed. Finally, this method is applied to two types of aluminum alloy, LC9 and LY12 for low-cycle fatigue, and agreed well with the experiments.

## 1. Introduction

The failure of machinery under cyclic loading is a major engineering concern in practice, and the fatigue properties of metal has long been a focus [1,2]. Many models were established by using equivalent stress [3,4,5], J-integral [5] and strain energy density [6,7] as a failure criterion. Among them, the strain energy density criterion has attracted much attention because of its wide applicability.

On one hand, the use of the energy theory to study the fatigue goes back a long way. Most of the works have been conducted based on the total strain energy density (TSED), which has been widely used to evaluate material failure [8]. Oliferuk and Maj [9] described the energy storage during the plastic deformation based on the experimental results, and gave the corresponding mathematical expression of the plastic strain energy. Shahrooi et al. [10] studied the damage criterion based on plastic strain energy for ratcheting under multiaxial loading and predicted the fatigue lives with compression to experimental data. Wang et al. [11] introduced the main fatigue life prediction models, including the Manson–Coffin formula, Basquin formula and the strain damage model. It was pointed out that the coefficients of the fatigue life prediction formulae were dependent on the material properties. Therefore, the constant coefficients used in the Manson–Coffin formula and the strain damage model was not reliable. Sun et al. [12] carried out the fatigue test of the nickel alloy GH4169 at 650 °C under the combination of proportional and non-proportional tension and torsion loadings, thereby establishing the damage model which could be simplified to the uniaxial Manson–Coffin equation. Xu et al. [13] proposed a damage evolution model for low-cycle fatigue considering that the fatigue damage accumulation is mainly caused by cyclic plastic strain. Martins et al. [14] studied the low-cycle fatigue life of bainitic steels based on the cumulative strain energy density and developed a new predictive model to estimate the fatigue life. Roy and Song et al. [15,16] gave the expression of plastic strain energy for Masing material under cyclic loading. Then, the plastic strain energy density was derived both analytically and experimentally for different materials. From these works, the fatigue damage in low cycle fatigue is mainly caused by the accumulation of cyclic plastic strain energy. However, the application of the incremental plastic strain energy density in metals’ failure analyses has not been reported in literature.

On the other hand, scholars have devoted much effort to the study of the fatigue damage process in terms of energy conversion and energy dissipation [17]. The Bauschinger effect and hysteresis phenomenon observed in fatigue have proven the energy dissipation in this process [18,19]. Azadi et al. [20] presented a lifetime prediction model based on the plastic strain energy for aluminum alloys. They performed thermal and mechanical fatigue tests using the A356 alloy. Vidal et al. [21] discussed the influence of the geometry on the bending strength and fatigue behavior of aluminum alloy specimens. As well as the fatigue lives being analyzed with the finite element method, Skibicki and Pejkowski [22] analysed the fatigue lives of CuZn37 based on the hysteresis loops energy in the loading cycle and the total plastic strain energy in the fatigue test. The relations between the plastic strain energy and the total plastic strain were described, too. Feng et al. [23] established an energy dissipation-based multiaxial fatigue model that allows the fatigue life can be assessed for a given strain path. It demonstrated that the energy dissipation-based method can provide satisfactory life prediction for the varied loading paths for both proportional and non-proportional loading. Aid et al. [24] proposed a non-linear model to estimate the fatigue damage under random load and the fatigue life of structural members. The proposed model was tested using 6082T6 aluminum alloy. The fatigue damage estimation of this model is better than the commonly used Palmgren–Miner rule. By introducing the non-linear stress-strain relationship, the deformation energy theory is applied to calculate the accumulation plastic strain energy under cyclic loading. Maurel et al. [25] analyzed the result of the symmetrical tension and compression test of some notched specimens of cylindrical ferritic stainless steel pipe. The mode of propagation of an artificial crack in low-cycle fatigue is given. Rozumek [26] summarized the models of the fatigue crack growth rate. Most of the energy approaches are based on the J-integral or the strain-energy density and corresponds to the entire range of the crack growth rate. Vormwald [27] discussed various proposals of crack driving force parameter in elastoplastic fracture and analyzed the consequences for fatigue lives under multiaxial loading with variable amplitude. Huffman [28] proposed a strain energy-based fatigue damage model and applied the strain energy from both the external loading and the dislocations to calculate stress-life, strain-life, and fatigue crack growth rates. Based on the critical plastic dissipation energy, Wang et al. [29] carried out numerical simulation of crack propagation. The results show that the fatigue crack propagation accords with the fatigue cumulative damage of material. In addition, the fatigue of metals considering the microstructures are studied from the perspective of energy dissipation [30,31]. Using the energy-based approach to study the fatigue problem has a long history. But due to the complexity of fatigue problems, the quantitative description of material energy storage is still quite difficult and needs further improvement.

Although many models have been presented to predict fatigue life of metals, there are still two imperfections to be overcome. First, the accuracy of those models is dependent on material properties and loading conditions. It is difficult to give a simple model to predict low cycle fatigue life. Second, those models are far from perfect especially in using the plastic strain energy density to establish relation between uni-axial tensile fracture and fatigue fracture. In this paper, the accumulated plastic strain energy density is applied to analyze the fatigue life for low cycle fatigue from the point of view of energy dissipation. Starting from the perspective of plastic energy accumulation, the plastic strain energy density of the dangerous point is used as the criterion to establish the relationship between uni-axial tensile fracture and fatigue failure. Then the fatigue life estimation formula is obtained and is used to predict the fatigue life.

## 2. Plastic Strain Energy Density under Uni-Axial Tension

An accurate description of the constitutive relation is critical for the calculation accuracy of the plastic strain energy density. Although, factors like microstructure [32], temperature [33] and strain rate [34] have confirmative influence on the relation between stress and strain, the macroscopic phenomenological models are still popular. Among these constitutive relations, the Ramberg–Osgood model [35] is widely used. Hertelé et al. [36] modified the Ramberg–Osgood constitutive model and proposed a constitutive model which was applicable to both aluminum alloy and stainless steel. Their study revealed that the modified model could be applied to other non-linear metallic materials with different parameters.

### 2.1. Modified Ramberg–Osgood Constitutive Equation

**Figure 1:**
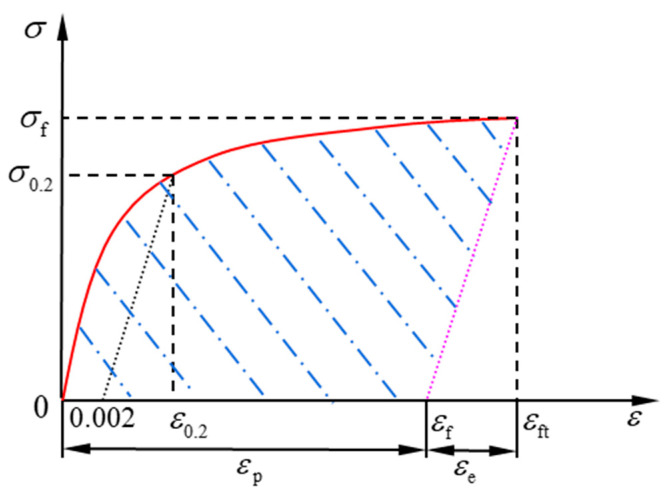
Non-linear stress–strain curve.

The modified Ramberg–Osgood constitutive relation could more precisely describe the nonlinear stress-strain relationship of strain-hardened ductile metals. The stress-strain curve is shown in Figure 1.

The modified Ramberg–Osgood stress–strain relation is expressed as follows:

(1) $$\varepsilon =\{\begin{array}{cc} \frac{\sigma }{E_{0}}+0.002{\left(\frac{\sigma }{\sigma_{0.2}}\right)}^{1/n} & 0\leq \sigma \leq \sigma_{0.2}\\ \frac{\sigma -\sigma_{0.2}}{E_{0.2}}+\left(\varepsilon_{f}-\varepsilon_{0.2}\right){\left(\frac{\sigma -\sigma_{0.2}}{\sigma_{f}-\sigma_{0.2}}\right)}^{m}+\varepsilon_{0.2} & \sigma_{0.2}<\sigma \leq \sigma_{f} \end{array}$$

where *E*_0_ is the elastic modulus, *E*_0.2_ is tangent modulus at *σ*_0.2_ which is the nominal yield stress, *n* is the strain hardening index, *ε*_0.2_ is the total strain at yielding, *σ*_f_ is the true stress upon the damage of material under monotonic loading, *ε*_f_ is the true fracture strain under monotonic loading, *m* is the shape correction parameter of the stress–strain curve. *m* is related to the yield strength and fracture strength. According to the literature [35], the value of m may be taken as:

(2) $$m=1+3.5\frac{\sigma_{0.2}}{\sigma_{f}}$$

The stress–strain relation expressed in Equation (1) could be presented by the curve shown in Figure 1 where *ε*_e_ is elastic strain, *ε*_p_ is the plastic strain, *ε*_ft_ is the total fracture strain.

### 2.2. Plastic Strain Energy Density

The plastic strain energy density (shaded area) should be the total strain energy minus the elastic strain energy density (the triangle on the right), as shown in Figure 1. Based on the constitutive equations Equations (1) and (2), the strain is used as the variable of integration. The accumulated plastic strain energy density at fracture under uni-axial tensile loading could be obtained.

The loading curve is expressed by Equation (1). The unloading curve is *ε* = *σ*/*E*_0_ + *ε*_f_. Since the strain is taken as the variable of integration, Equation (1) needs to be rewritten as the formula of stress in terms of strain. To do that, the strain is divided into elastic and plastic parts. Then the stress could be written in terms of plastic strain:

(3) $$\sigma =\{\begin{array}{cc} \sigma_{0.2}{\left(\frac{\varepsilon_{p}}{0.002}\right)}^{n} & 0\leq \varepsilon_{p}\leq 0.002\\ \left(\sigma_{f}-\sigma_{0.2}\right){\left(\frac{\varepsilon_{p}-0.002}{\varepsilon_{f}-0.002}\right)}^{1/m}+\sigma_{0.2} & 0.002<\varepsilon_{p}\leq \varepsilon_{f} \end{array}$$

**Figure 2:**
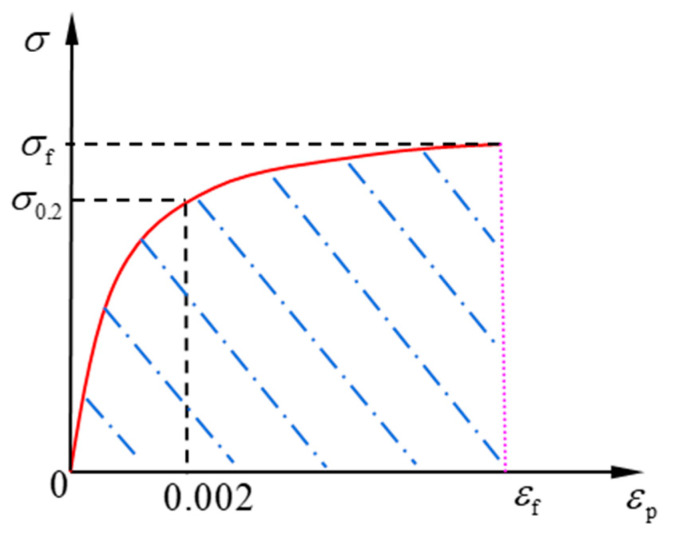
Relationship between stress and plastic strain.

The plastic strain and stress relationship is shown in Figure 2, where the shaded area is the plastic strain energy density that could be calculated by integrating Equation (3) with respect to strain, as shown in Equation (4):

(4) $$\begin{array}{ll} W_{f} & =\int_{0}^{0.002}d\varepsilon_{p}\int_{0}^{\sigma_{0.2}{\left(\frac{\varepsilon_{p}}{0.002}\right)}^{n}}d\sigma +\int_{0.002}^{\varepsilon_{f}}d\varepsilon_{p}\int_{0}^{\left(\sigma_{f}-\sigma_{0.2}\right){\left(\frac{\varepsilon_{p}-0.002}{\varepsilon_{f}-0.002}\right)}^{1/m}+\sigma_{0.2}}d\sigma \\ & =\int_{0}^{0.002}\left[\sigma_{0.2}{\left(\frac{\varepsilon_{p}}{0.002}\right)}^{n}\right]d\varepsilon_{p}+\int_{0.002}^{\varepsilon_{f}}\left[\left(\sigma_{f}-\sigma_{0.2}\right){\left(\frac{\varepsilon_{p}-0.002}{\varepsilon_{f}-0.002}\right)}^{1/m}+\sigma_{0.2}\right]d\varepsilon_{p}\\ & =\frac{0.002}{n+1}\sigma_{0.2}+\frac{m}{m+1}\left(\sigma_{f}-\sigma_{0.2}\right)\left(\varepsilon_{f}-0.002\right)+\sigma_{0.2}\left(\varepsilon_{f}-0.002\right) \end{array}$$

Two metals, LY12 and LC9, are used for subsequent theoretical analyses. The materials’ parameters are listed in Table 1. By applying the method mentioned above, the parameters of the constitutive relation and plastic strain energy density under uniaxial tension could be obtained.

In order to calculate the plastic strain energy density of the dangerous point, *ε*_0.2_, *E*_0.2_, *m* and other parameters should be determined first according to Equation (1). The first two could be get directly by *E*_0.2_ = d*σ*/d*ε* while *σ* = *σ*_0.2_ and *ε*_0.2_ = *σ*_0.2_/*E*_0.2_ + 0.002. *m* is obtained from Equation (2). These parameters and the plastic strain energy density of the two materials are listed in Table 2.

## 3. Accumulated Plastic Strain Energy Density under Cyclic Loading

**Figure 3:**
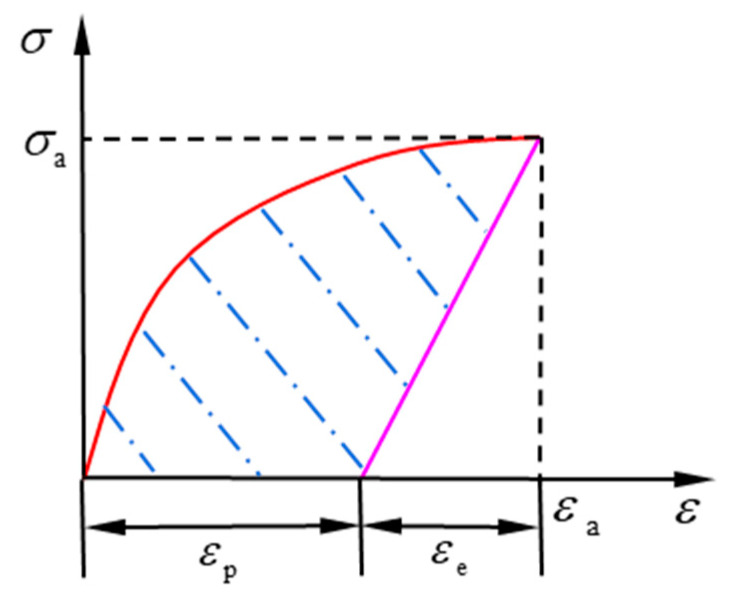
Stress–strain curve of a single cycle.

The plastic strain energy density accumulated at the dangerous point within a single cycle under uniaxial cyclic loading is considered. When applying the modified Ramberg–Osgood model, the unloading curve can be formulated by *ε* = (*σ* − *σ*_a_)/*E*_0_ + *ε*_a_, as shown in Figure 3. The shaded area is the plastic strain energy density accumulated within one cycle.

Then, the formula of single-cycle plastic strain energy density is expressed as follows:

$$0\leq \varepsilon_{\mathrm{pa}}\leq 0.002,$$

(5) $$\Delta W_{p}=\int_{0}^{\varepsilon_{\mathrm{pa}}}{\sigma^{\prime }}_{0.2}{\left(\frac{\varepsilon_{\mathrm{pa}}}{0.002}\right)}^{n^{\prime }}d\varepsilon_{\mathrm{pa}}=\frac{{\sigma^{\prime }}_{0.2}}{{\left(0.002\right)}^{n^{\prime }}}\frac{1}{n^{\prime }+1}{\left(\varepsilon_{\mathrm{pa}}\right)}^{n^{\prime }+1}$$

when,

$$0.002<\varepsilon_{\mathrm{pa}}\leq {\varepsilon^{\prime }}_{f},$$

(6) $$\begin{array}{ll} \Delta W_{p} & =\int_{0}^{0.002}{\sigma^{\prime }}_{0.2}{\left(\frac{\varepsilon_{\mathrm{pa}}}{0.002}\right)}^{n^{\prime }}d\varepsilon_{\mathrm{pa}}+\int_{0.002}^{\varepsilon_{\mathrm{pa}}}\left[\left({\sigma^{\prime }}_{f}-{\sigma^{\prime }}_{0.2}\right){\left(\frac{\varepsilon_{\mathrm{pa}}-0.002}{{\varepsilon^{\prime }}_{f}-0.002}\right)}^{1/m^{\prime }}+{\sigma^{\prime }}_{0.2}\right]d\varepsilon_{\mathrm{pa}}\\ & =\frac{0.002{\sigma^{\prime }}_{0.2}}{n^{\prime }+1}+\frac{m^{\prime }}{m^{\prime }+1}\frac{\left({\sigma^{\prime }}_{f}-{\sigma^{\prime }}_{0.2}\right)}{{\left({\varepsilon^{\prime }}_{f}-0.002\right)}^{1/m^{\prime }}}{\left(\varepsilon_{\mathrm{pa}}-0.002\right)}^{1+1/m^{\prime }}+{\sigma^{\prime }}_{0.2}\left(\varepsilon_{\mathrm{pa}}-0.002\right) \end{array}$$

where *ε*_pa_ is the plastic strain amplitude of the unloading/reloading. *σ*’_f_ is the fatigue strength coefficient. *ε*’_f_ is the fatigue ductility coefficient. *m*’ is the shape parameter under cyclic loading. *σ*’_0.2_ is the yield stress under cyclic loading. *ε*’_0.2_ is the total strain under cyclic loading when material yield. *E*’_0.2_ is tangent modulus at *σ*’_0.2_.

To calculate the plastic strain energy density accumulated in one cycle for the two materials LY12 and LC9, the fatigue parameters required are listed in Table 3.

In order to apply the modified Ramberg–Osgood stress–strain relation to calculate the plastic strain energy density, the same method should be adopted for the parameters of Equations (5) and (6) as in the previous section:

(7) $${\varepsilon^{\prime }}_{0.2}=\frac{{\sigma^{\prime }}_{0.2}}{E_{0}}+0.002,\;{E^{\prime }}_{0.2}=\frac{E_{0}}{1+\frac{0.002E_{0}}{n^{\prime }{\sigma^{\prime }}_{0.2}}},\;m^{\prime }=1+3.5\frac{{\sigma^{\prime }}_{0.2}}{{\sigma^{\prime }}_{f}}$$

The results are listed in Table 4.

According to the fatigue parameters of the two materials and calculation parameters given by Table 4, Equation (6) could be used to calculate the accumulated plastic strain energy densities of a single cycle for the two materials above under the cyclic loading. The results are listed in Table 5.

## 4. Fatigue Life Estimation

In this paper, we try to establish a method for low cycle fatigue life assessment from the energy dissipation point of view; that is, along with the energy dissipation caused by plasticity, the material damages and fractures. For this purpose, two hypotheses should be addressed:

(1) Accumulated plastic strain energy density causes the damage and fracture of material;
(2) Material damages or fractures when the accumulated plastic strain energy density reaches a critical value.

From here on, these hypotheses are applied to assess the fatigue life of metals. The critical value of the accumulated plastic strain energy density could be obtained by uni-axial tension. Despite the plastic strain energy densities under the uni-axial tension and cyclic loading are different, they are related to each other closely since both of them represent the energy dissipation and material degradation. It should be pointed out that the fatigue failure study does not mean the fracture of the whole material, but the failure at the dangerous point, that leads to the material separation (or forming micro cracks). Therefore, the linear cumulative damage theory based on plastic strain energy density is used to predict the fatigue life of the material under cyclic loading.

The process of fatigue is divided into two stages, fatigue crack initiation and fatigue crack propagation. The first stage is the process from damage (micro crack nucleation) to a macro crack formed, and the second stage is the crack stable propagation to final fracture.

### 4.1. Estimation of Crack Initiation Life

With applying the modified Ramberg–Osgood relationship, the plastic strain energy density in a single cycle could be formulated from Equation (5) as:

(8) $$\varepsilon_{\mathrm{pa}}={\left(\frac{1+n^{\prime }}{{\sigma^{\prime }}_{0.2}}{\left(0.002\right)}^{n^{\prime }}\Delta W_{p}\right)}^{\frac{1}{1+n^{\prime }}}$$

From Equation (8), the relation between fatigue life and plastic strain range could be established as follows:

(9) $$\Delta \varepsilon_{p}\cdot N^{\beta }=2{\left(\frac{1+n^{\prime }}{{\sigma^{\prime }}_{0.2}}{\left(0.002\right)}^{n^{\prime }}\Delta W_{p}\right)}^{\frac{1}{1+n^{\prime }}}N^{\beta }=C$$

where *N* is the number of cycle, Δ*ε*_p_ is the change of plastic strain. Equation (9) is derived by applying the linear damage accumulation principle which considers the plastic strain energy density accumulated in every cycle is a constant. Furthermore, when subjected to cyclic loading with non-zero mean stress, the stress–strain curve of material in one cycle is almost antisymmetric, indicating the area enclosed in a loop’s curve is almost twice the area above the mean stress. This is the reason why the *ε*_pa_ is replaced by 2 × Δ*ε*_p_ in Equation (9). Then the relation between the plastic strain energy density and the number of cycles is expressed as follows:

(10) $$\Delta W_{p}=\frac{{\sigma^{\prime }}_{0.2}}{\left(1+n^{\prime }\right){\left(0.002\right)}^{n^{\prime }}}{\left(\frac{C}{2N^{\beta }}\right)}^{1+n^{\prime }}=\frac{{\sigma^{\prime }}_{0.2}}{\left(1+n^{\prime }\right){\left(0.002\right)}^{n^{\prime }}}{\left(\frac{C}{2}\right)}^{1+n^{\prime }}N^{-\beta \left(1+n^{\prime }\right)}$$

It can be seen from the above equation that the cyclic plastic strain energy density range has an exponential relationship with the fatigue life, which is denoted as:

(11) $$\Delta W_{p}=\alpha \cdot N^{b}$$

where *α* and *b* are constants.

Equation (11) shows the relationship between the single cycle plastic strain energy density and fatigue life. 

According to the linear damage accumulation principle that assumes the plastic strain energy density accumulated in every cycle is not changed with the increase of cycle times:

(12) $$W_{p}=\Delta W_{p}\cdot N$$

Substituting Equation (11) to Equation (12):

(13) $$W_{p}=\alpha \cdot N^{b+1}$$

Meanwhile, the total plastic strain energy density under the uni-axial tension could be regarded as a special case of fatigue loading, that is, the corresponding total plastic strain energy density when the fatigue life is 1. Thus, constant *α* should be equal to the plastic strain energy density calculated in the uni-axial tension conditions in this article. Then the constants *b* could be fitted from Equation (11).

### 4.2. Estimation of Crack Propagation Life

For brittle materials, the fracture criterion is given by the Griffith criterion.

(14) $$\sigma_{f}=\sqrt{\frac{2E\gamma }{\pi a}}$$

where, *σ_f_* is the nominal fracture stress, *γ* is the surface free energy and *a* is the crack length. The elastic strain energy should decrease accompanying the crack propagating and the reduction of the elastic strain energy should reach the energy needed to form a new crack surface at least. 

If the specimen is subjected to two different stress aptitudes *σ*_1_ and *σ*_2_, respectively, the relation of critical crack length *a*_1_ and *a*_2_ can be deduced from Equation (14) as:

(15) $$\frac{a_{1}}{a_{2}}={\left(\frac{\sigma_{2}}{\sigma_{1}}\right)}^{2}$$

When the stress is small, the final fracture of fatigue is caused by a longer crack in advance, and vice-versa.

Equation (14) describes a fracture criterion not only for fatigue loading but also for uni-axial tensile loading. However, the mechanism and the process of reaching breaking point is essentially different. Under uni-axial tensile loading, the stress increases gradually before the fracture occurs. While under fatigue loading, the stress increases due to the gradual expansion of the crack, and the bearing area is decreased. This indicates that crack propagation can produce the critical stress causing the final fracture. In the following discussion, the cyclic plastic strain energy will be used as a criterion for crack propagation.

There exists a plastic zone at the crack tip if the material with a crack is loaded. The energy required for the plastic deformation near the crack depends on the volume of the plastic zone and the distribution of the strain in the plastic zone. It is assumed that the volume of the plastic deformation zone is proportional to *γ* sub square of the crack length. Then, when two cracks with different length expand, the relationship between the plastic strain energy density required by the expansion is [38]:

(16) $$\frac{W_{p1}}{W_{p2}}={\left(\frac{a_{1}}{a_{2}}\right)}^{\gamma }$$

To simplify the fatigue problems, the following approximate simplification is proposed in this paper. Let *σ*_f_ and *W*_f_ represent the true fracture stress and critical plastic strain energy density corresponding to the uniaxial tensile fracture, respectively. *σ_a_* and *W_p_* denote the cyclic stress amplitude and the total cyclic plastic strain energy density corresponding to the fatigue fracture, respectively. From Equations (15) and (16), we have:

(17) $$\frac{\sigma_{a}}{\sigma_{f}}={\left(\frac{W_{p}}{W_{f}}\right)}^{-\frac{1}{2\gamma }}$$

For the calculation of total plastic strain energy density under fatigue fracture, the linear damage accumulation principle is adopted. It is assumed that the plastic strain energy density accumulated in every cycle is not changed with the increase of cycle times, that is, the hysteresis loops of the material at different cycles are the same. The cumulative plastic strain energy density of each cycle is Δ*W*_p_. The total cyclic plastic strain energy density is:

(18) $$W_{p}=\Delta W_{p}\cdot N_{f}$$

By substituting Equation (17) into Equation (18), the formula of the fatigue crack growth life can be obtained:

(19) $$N_{f}=\frac{W_{f}}{\Delta W_{p}}{\left(\frac{\sigma_{a}}{\sigma_{f}}\right)}^{-2\gamma }$$

According to the modified Ramberg–Osgood constitutive relation, the plastic strain energy density of a single cycle is Δ*W*_p_, as shown in Equations (5) and (6). The total cyclic plastic strain energy density *W*_f_ can be obtained based on Equation (4). Then the life of fatigue crack growth can be obtained by Equation (19).

For a given material, the *γ* value could be determined. Then the fatigue life of materials under the given stress is estimated. According to Equation (17), *σ*_a_-*W*_p_ line could be drawn on the double logarithm coordinate to check and calculate the value of *γ*. The line passes through *W*_f_ and *σ*_f_ point, its slope equal to the exponent of the equation, −1/2*γ*. For most metals, the slope is between −1/8 and −1/3 [38].

### 4.3. Examples

**Figure 4:**
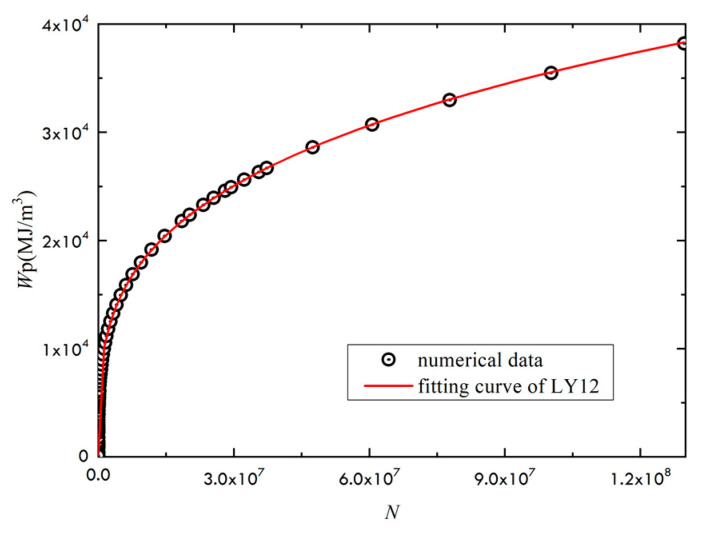
Relation between the accumulated plastic strain energy density and fatigue life of LY12.

**Figure 5:**
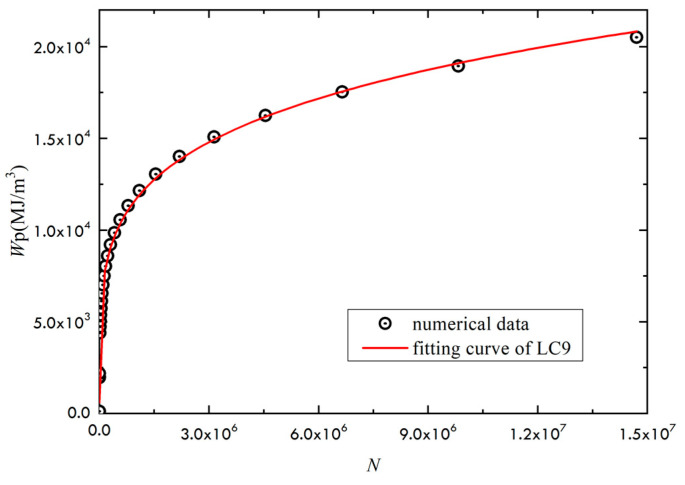
Relation between the accumulated plastic strain energy density and fatigue life of LC9.

The modified Ramberg–Osgood stress–strain relationship is used in the study. Given the stress ratio of 0, the fitting is conducted from the relationship between fatigue life and accumulative plastic strain energy density for the two materials LY12 and LC9, as shown in Figure 4 and Figure 5. The constants *α* and *b* could be numerically fitted. By fitting, *α* = 106.94 and *b* + 1 = 0.2927 could be obtained for LY12 material, and *α* = 200.42, *b* + 1 = 0.2147 for LC9 material.

According to the known parameters of LY12 and LC9, the relationship between stress and the total accumulated plastic strain energy density could be fitted. These parameters conform to the linear relation in the double logarithmic coordinates. The fitted constant *γ* is expressed as: −1/2*γ* = −0.2105 for LY12, and −1/2*γ* = −0.3348 for LC9. By substituting these parameters into Equation (19), the crack propagation life of the corresponding material could be obtained.

According to the *α*, *b* and *γ* determined above, Equations (11) and (19) are used to predict the fatigue crack initiation life and propagation life under different loading conditions, with the results shown in Table 6 and Table 7.

To verify the validation of current approach, experimental results are necessary.

### 4.4. Experimental Verification

The first fatigue test was carried out on the LC9 aluminum alloy using the PA-100 electro-hydraulic servo fatigue testing machine. The specimens were machined to the funnel-shaped round bar. The loading conditions were 8 ± 4 kN, 8 ± 5 kN and 8 ± 6 kN, respectively. The maximum values of *σ*_max_ loaded were 71.12%, 77.04% and 82.97% of the yield stress *σ*_0.2_, respectively. The loading frequency was 10 Hz. According to the loading and unloading curves in the experiment, the stress–strain curve within one cycle is used to calculate the enclosed area, and thus the accumulated plastic strain energy in one cycle is obtained. The formula established in this paper was used to predict the fatigue life and compared with the experimental results. The results are shown in Table 8.

The second fatigue test was carried out on the LY12 aluminum alloy. The loading conditions were 6 ± 4 kN, 6 ± 5 kN and 6 ± 6 kN, respectively. The maximum values of *σ*_max_ loaded were 73.75%, 81.04% and 88.54% of the yield stress *σ*_0.2_, respectively. The comparison between the proposed method and the experimental results is shown in Table 9.

The data in Table 8 and Table 9 indicate that the fatigue life estimated by the formula of this article is close to the experimental value. The calculation and experimental error is caused by the factors such as internal defects of materials. The fatigue life estimated by the formula of the current model is closer to the experimental value compared with the result by the Manson formula.

Table 10 and Table 11 show the calculated and experimental values of the fatigue life of LC9 and LY12 under the same stress amplitude for different average stress. The data in the table show that different average stresses have a greater influence on the fatigue life.

In general, the predicted results agree well with the experiments. The average errors between the proposed method and experiments are 14.7% and 20.6% in Table 10 and Table 11, respectively. In addition, the experimental results are always smaller than calculated values. It can be understood that there must exist some defects or inhomogeneities in the test specimen, and surface defects from manufacturing are hard to avoid.

## 5. Conclusions

In this paper, a model to assess the low cycle fatigue life based on the accumulated plastic strain energy density is proposed. In this model fatigue is divided into two stages, fatigue initiation and fatigue propagation. Both stages are evaluated by the accumulated plastic strain energy density. The experimental results agree well with the current model on fatigue life, implying the validity of this model.

In particular, the threshold value of accumulated plastic strain energy density is defined by the static tensile experiment, bridging the fatigue life to a simple standard test and clarifying the threshold value of the fatigue parameter which has long been a thorny issue. The results confirm the feasibility of this hypothesis.

## Figures and Tables

**Table 1:** The mechanical parameters for two materials [37].

Materials	*E*_0_ (MPa)	*σ*_0.2_ (MPa)	*σ*_f_ (MPa)	*ε*_f_ (%)	*n*
LY12	73,160.2	399.5	643.44	18	0.158
LC9	72,179.5	518.2	748.47	28.34	0.071

**Table 2:** *ε*_0.2_, *E*_0.2_, *m* and plastic strain energy densities.

Material Parameters	*ε*_0.2_ (%)	*E*_0.2_ (MPa)	*m*	*W*_f_ (MJ/m^3^)
LY12	0.75	22,049	3.2794	105.0757
LC9	0.92	14,660	3.4232	196.9376

where *ε*_0.2_ is the yielding strain, *E*_0.2_ is the tangent modulus at *σ*_0.2_, *m* is the shape correction parameter.

**Table 3:** Fatigue parameters of LY12 and LC9 [37].

Materials	*E*_0_ (MPa)	*σ*’_0.2_ (MPa)	*σ*’_f_ (MPa)	*ε*’_f_ (%)	*n*’
LY12	73,160.2	480.42	723.76	13.67	0.097
LC9	72,179.5	518.2	807.8	77.08	0.101

**Table 4:** *ε’*_0.2_ (yielding strain under cyclic loading), *E’*_0.2_ (tangent modulus at *σ’*_0.2_) and *m’* (shape parameter under cyclic loading) of LY12 and LC9.

Materials	*ε*’_0.2_ (%)	*E*’_0.2_ (MPa)	*m*’
LY12	0.86	17559	3.3037
LC9	0.92	19206	3.2452

**Table 5:** Plastic strain energy densities of LY12 and LC9 in a single cycle.

Materials	LY12			LC9		
Cyclic strain *ε*_pa_ (%)	0.01	0.2	1	0.01	0.2	1
Cyclic stress *σ*_a_ (MPa)	359.27	480.42	584.47	382.91	518.20	589.13
Plastic strain energy density (MJ/m^3^)	0.0328	0.8759	5.3549	0.0348	0.9413	5.5207

**Table 6:** Life estimation of LY12.

*ε*_pa_ (%)	*σ*_a_ (MPa)	Δ*W*_p_ (MJ/m^3^)	Fatigue Crack Initiation Life	Fatigue Crack Propagation Life	Fatigue Life
0.002	307.34	0.0056	1,127,623	627,293	1,754,916
0.01	359.27	0.0328	92,914	51,121	144,035
0.2	480.42	0.8759	892	481	1310
1	584.47	5.3549	69	31	100

**Table 7:** Life estimation of LC9.

*ε*_pa_ (%)	*σ*_a_ (MPa)	Δ*W*_p_ (MJ/m^3^)	Fatigue Crack Initiation life	Fatigue Crack Propagation Life	Fatigue Life
0.002	325.46	0.0059	587,154	400,727	987,881
0.01	382.91	0.0348	61,488	41,919	103,407
0.2	518.20	0.9413	922	627	1549
1	589.13	5.5207	97	73	170

**Table 8:** Estimated and experimental results of the fatigue life of LC9.

Loading	8 ± 4 kN	8 ± 5 kN	8 ± 6 kN
Δ*W*_p_ (MJ/m^3^)	0.0205	0.0642	0.1270
Proposed method	173,067	41,283	17,154
Manson formula [11]	208,270	39,940	18,627
Experimental results	169,706	35,184	15,653

**Table 9:** Estimated and experimental results of the fatigue life of LY12.

Loading	6 ± 4 kN	6 ± 5 kN	6 ± 6 kN
Δ*W*_p_ (MJ/m^3^)	0.0277	0.0805	0.219
Proposed method	179,604	39,716	9616
Manson formula [11]	190,325	40,087	10,532
Experimental results	128,991	36,598	7,699

**Table 10:** Estimated and experimental results of the fatigue life of LC9 for different average stresses (the stress amplitude is 5 kN).

Average Stresses	5 kN	7 kN	8 kN	10 kN
Δ*W*_p_ (MJ/m^3^)	0.0148	0.0236	0.0642	1.2375
Proposed method	303,465	141,019	41,283	2125
Experimental results	274,679	122,192	35,184	1834

**Table 11:** Estimated and experimental results of the fatigue life of LY12 for different average stresses (the stress amplitude is 5 kN).

Average Stresses	5 kN	6 kN	7 kN	8 kN
Δ*W*_p_ (MJ/m^3^)	0.0277	0.0.805	0.2190	0.5359
Proposed method	179,604	39,716	9616	2708
Experimental results	113,769	36,598	8506	2625
